# Development and validation of a predictive nomogram for subsequent contralateral hip fracture in elderly patients within 2 years after hip fracture surgery

**DOI:** 10.3389/fmed.2023.1263930

**Published:** 2023-12-21

**Authors:** Jiahui Liang, Jian Zhang, Zhiyuan Lou, Xin Tang

**Affiliations:** ^1^Department of Orthopedics, First Affiliated Hospital of Dalian Medical University, Dalian, China; ^2^Graduate School of Dalian Medical University, Dalian Medical University, Dalian, China

**Keywords:** contralateral hip fracture, prediction, nomogram, elderly patients, hip fracture surgery

## Abstract

**Purpose:**

Contralateral hip refracture following initial hip fracture surgery is life-threatening in the elderly with high incidence and mortality. This study investigated the associated independent risk factors and established a nomogram prediction model.

**Methods:**

Totally 734 elderly patients with hip fractures who underwent surgical treatment (January 2016–December 2020) were enrolled. Following analyses on demographic variables, clinical characteristics, and laboratory examination, independent risk factors of contralateral hip fractures in the elderly were identified through the least absolute shrinkage and selection operator (LASSO) regression, and univariate and multivariate logistic regression. Patients were randomly allocated into training (*n* = 513) and validation sets (*n* = 221). A training set-based nomogram prediction model was established and assessed for predictability, discriminatory ability, and clinical applicability using the receiver operating characteristic (ROC) curves, calibration curves, and decision curve analysis (DCA) in both sets.

**Results:**

Contralateral hip refractures occurred in 7.08% (52/734) patients within 2 years after surgery. Age, hemoglobin (Hb), heart disease, neurovascular disease, Parkinson’s disease (PD), Alzheimer’s disease (AD), chronic obstructive pulmonary disease (COPD), and chronic kidney disease (CKD) were independent risk factors. The nomogram prediction model had a favorable discriminatory ability, as indicated by the areas under the ROC curves (AUC): 0.906 (95% CI, 0.845–0.967) in the training set and 0.956 (95% CI, 0.927–0.985) in the validation set. The calibration curves demonstrated a good consistency between the actual subsequent contralateral hip fracture incidence and the predicted probability. The DCA of the nomogram demonstrated the model’s excellent clinical efficacy.

**Conclusion:**

The nomogram model enabled accurate individualized prediction for the occurrence of subsequent contralateral hip fracture in the elderly within 2 years after surgical treatment, which might help clinicians with precise references for appropriate perioperative management and rehabilitation education following initial hip surgery for their patients.

## 1 Introduction

Elderly osteoporotic hip fractures categorized as femoral neck and intertrochanteric fractures in accordance with the location were associated with significant mortality and extensive medico-economic burden (1). Prior osteoporotic fractures were considered a robust predictor of subsequent fractures, with a relative risk of approximately 2.7-fold (2). As previously reported, the subsequent contralateral hip fracture has an incidence of 4.4%–17.9% (3–5), occurring primarily within 2 years following surgical treatment of the previous hip fracture (6, 7).

Subsequent hip fractures involved prolonged hospitalization and rehabilitation, severely diminishing the quality of life, accompanied by some other consequences (disability, cardiovascular diseases, etc.) (8, 9). Furthermore, previous literature revealed that patients with subsequent hip fractures presented an additional 55% higher mortality risk than those without subsequent hip fractures (10).

Targeted prevention interventions contribute positively to reducing the occurrence of subsequent hip fractures, enhancing patients’ life quality and alleviating the socioeconomic burden (9, 11). In recent years several clinical investigations focused on exploring the subsequent contralateral hip fractures-associated risk factors including age, gender, cardiovascular disease, visual impairment, neurovascular disease, COPD, and AD (12–14). However, all the above research neglected the effect of laboratory examination indicators on subsequent contralateral hip fractures. Additionally, it remains a lack of research studies that translate the associated risk factors into practical risk scales or predictive models.

Nomogram, a visual way to present multifactorial predictive models, can simplify models to a single numerical estimate of event probabilities, which shows a wide application in orthopedic clinical practice (15–18). Therefore, our study aimed to establish a nomogram model for the contralateral hip fracture postoperatively that can comprehensively analyze its independent risk factors from demographic statistics, clinical characteristics, and laboratory examination indicators, which contribute to the individualized prediction of refracture risks within 2 years after fracture surgery in the elderly. This study may provide a reference for early intervention for improving outcomes.

## 2 Materials and methods

### 2.1 Patient

This retrospective study involved elderly hip fracture patients who underwent surgical intervention in our level-1 trauma center between January 2016 and December 2020. The inclusion criteria were: age ≥ 60 years; low energy damage-induced initial hip fracture (diagnosed as femoral neck fracture and intertrochanteric fracture with radiological and clinical symptoms); unilateral fresh closed fracture (injury-to-surgery time < 3 weeks); surgical treatment includes joint replacement, open reduction and closed reduction internal fixation; the clinical data were completed. The exclusion criteria were: hip fractures due to diseases such as joint tuberculosis and tumors; multiple fractures or multiple traumas; patients with previous hip arthroplasty or internal fixation of the hip; ipsilateral hip refracture (periprosthetic fracture) within 2 years; patients with blood system disorders or other disorders affecting blood tests; incomplete follow-up information owing to patient death (The death may be due to factors such as advanced age and severe complications, which may affect the expected outcome), etc. According to the requirements for developing a clinical prediction model, the sample size should be at least 10 times the number of variables (19, 20). In our study there were 38 variables, thus the sample size should be at least 380, and therefore our sample size is adequate.

This study got approval from the Ethics Committee of the First Affiliated Hospital of Dalian Medical University. The ethical approval number is PJ-KS-KY-2023-279. All the experiments were carried out following the Helsinki Declaration.

### 2.2 Data collection

Data (demographic variables, clinical characteristics, and laboratory examination) of all patients were collected. Demographic variables included age, gender, place of residence, height, weight, and body mass index (BMI). Clinical characteristics were hospitalization time, American Society of Anesthesiologists Physical Status Classification (ASA class), fracture type, surgical approach, hypertension, heart disease (coronary artery disease, heart failure, arrhythmia, heart dysfunction, chronic pulmonary heart disease), diabetes, neurovascular disease (encephalorrhagia, cerebral thromboembolism, cerebral infarction sequela), PD, AD, eye disease (cataract, glaucoma, diabetic eye disease), COPD, CKD, and chronic liver disease (chronic hepatitis, hepatic cirrhosis, hepatic insufficiency). Laboratory examination involved white blood cell (WBC), Hb, platelet (PLT), neutrophil (NEU), lymphocyte (LYM), neutrophil-to-lymphocyte ratio (NLR), platelet-to-lymphocyte ratio (PLR), systemic immune-inflammation index (SII; platelet count × neutrophil count/lymphocyte count), potassium, sodium, calcium, and phosphorus in serum, calcium-phosphorus product (serum calcium × serum phosphorus × 12.4), fasting blood glucose (FBG), albumin (ALB), alkaline phosphatase (ALP), creatinine (Cr), and blood urea nitrogen (BUN).

The blood samples were collected in the morning after admission, following an overnight fasting period. The data of demographic variables, clinical characteristics and comorbidities were acquired from the patient’s hospital records.

### 2.3 Development and validation of the prediction model

The (glmnet) package of R-studio 4.2.1 (R Foundation for Statistical Computing, Austria) was used for LASSO regression to screen out statistically significant variables for subsequent contralateral hip fracture. The independent risk factors were identified through univariate and multivariate logistic regression analysis on variables with non-zero coefficients. Patients were allocated (training set: validation set = 7:3) at random. The predictive nomogram model was established on the training set, and its performance was assessed by the validation set. The “rms” R package was utilized for nomogram construction and visualization. The ROC curve was applied to assess model discrimination. The calibration of the nomogram was evaluated by the Hosmer-Lemeshow (HL) goodness-of-fit test on the comparison of actual risk with predicted risk. Additionally, the DCA curve was conducted to assess the model’s clinical practicability.

### 2.4 Statistical analysis

SPSS 25.0 (IBM, Armonk, NY, United States) and R-studio software were applied for data analysis. For continuous variables, the normality was measured using the Shapiro–Wilk test. Normally distributed data were shown as mean ± standard deviation (SD), accompanied by independent Student’s t-test analysis, while those with non-normal distribution were described as the median and interquartile range M (IQR), accompanied by Mann–Whitney U test analysis. Categorical data were described as numbers and percentages (%), followed by the chi-square test or Fisher’s exact test analysis. A two-tailed *p* value of less than 0.05 indicated a significant difference.

## 3 Results

### 3.1 Patient baseline data

Totally 734 patients were enrolled. Among these patients, 52 patients suffered a contralateral hip refracture within 2 years postoperatively. The baseline demographic variables, clinical characteristics, and laboratory examination were summarized in Table 1. Patients were assigned to training and validation sets randomly. As shown in Table 2, no significant difference in all variables between the two sets was found except in PLR (*p* = 0.047), indicating that the training and validation sets were randomly assigned.

**Table 1:** Baseline characteristics of patients with or without subsequent contralateral hip fracture after initial hip fracture surgery.

Variable	Contralateral hip fracture (*n* = 52)	Initial hip fracture (*n* = 682)	Statistical value	*p* value
Gender (*n*, %)			2.932	0.099
Male	8 (15.4)	178 (26.1)		
Female	44 (84.6)	504 (73.9)		
Age (years)	82.23 ± 6.52	79 (70, 83)	−4.431	<0.001*
Hospitalization time (d)	9.5 (7, 13)	9.9 (7, 13)	−0.239	0.811
Place of residence (*n*, %)			0.768	0.409
City	42 (80.8)	514 (75.4)		
Rural	10 (19.2)	168 (24.6)		
ASA class (*n*, %)			19.442	<0.001*
1	0 (0)	6 (0.9)		
2	9 (17.3)	260 (38.1)		
3	34 (65.4)	382 (56)		
4	9 (17.3)	34 (5)		
Height (m)	1.55 (1.53, 1.64)	1.58 (1.55, 1.64)	−1.818	0.069
Weight (kg)	59.83 ± 9.94	60 (53, 68)	−0.158	0.875
BMI (kg/m^2^)	23.85 ± 3.62	23.4 (20.8, 26.7)	−0.478	0.632
Fracture type (*n*, %)			3.692	0.06
Intertrochanteric fracture	29 (55.8)	287 (42.1)		
Femoral neck fracture	23 (44.2)	395 (57.9)		
Surgical approach (*n*, %)			11.168	0.011*
Closed reduction internal fixation	29 (55.8)	284 (41.6)		
Hemiarthroplasty	17 (32.7)	166 (24.3)		
Total hip arthroplasty	4 (7.7)	166 (24.3)		
Open reduction internal fixation	2 (3.8)	66 (9.7)		
WBC (10^9^/L)	8.48 ± 2.28	8.11 (6.44, 9.73)	−0.675	0.499
Hb (g/L)	111 (93, 118)	121 (107, 133)	−4.619	<0.001*
PLT (10^9^/L)	178.48 ± 41.76	187.5 (155, 228.25)	−1.989	0.047*
NEU (10^9^/L)	6.83 ± 2.14	6.02 (4.62, 7.52)	−1.98	0.048*
LYM (10^9^/L)	1.10 (0.79, 1.31)	1.22 (0.92, 1.56)	−2.343	0.019*
NLR	6.35 (4.55, 10.27)	4.88 (3.32, 6.96)	−2.978	0.003*
PLR	174.65 (124.84, 229.87)	156.34 (115.18, 209.29)	−1.431	0.152
SII	1168.81 (730.3, 1667.35)	906.75 (593, 1432.74)	−2.204	0.028*
Calcium (mmol/L)	2.17 (2.07, 2.24)	2.2 (2.13, 2.27)	−2.157	0.031*
Phosphorus (mmol/L)	1.07 ± 0.23	1.11 (1, 1.25)	−2.142	0.032*
Calcium-phosphorus product	28.8 ± 6.44	30.26 (26.95, 34.62)	−2.223	0.026*
Potassium (mmol/L)	3.9 (3.59, 4.09)	3.8 (3.53, 4.1)	−0.328	0.743
Sodium (mmol/L)	139.07 ± 4.33	139 (136, 141)	−0.756	0.449
FBG (mmol/L)	6.29 (5.51, 7.24)	6.52 (5.68, 7.74)	−1.031	0.303
ALB (g/L)	35.42 ± 4.34	37.3 (34.48, 39.8)	−2.228	0.026*
ALP (U/L)	73 (63, 94.75)	77 (66, 96.25)	−0.875	0.382
Cr (μmol/L)	67.5 (52, 82.5)	60 (51, 74)	−1.776	0.076
BUN (mmol/L)	7.68 (5.99, 10.3)	6.09 (4.82, 7.76)	−3.673	<0.001*
Hypertension, yes (*n*, %)	32 (61.5)	315 (46.2)	4.568	0.043*
Heart disease, yes (*n*, %)	28 (53.8)	131 (19.2)	34.16	<0.001*
Diabetes, yes (*n*, %)	11 (21.2)	163 (23.9)	0.202	0.737
Neurovascular disease, yes (*n*, %)	26 (50)	106 (15.5)	38.894	<0.001*
PD, yes (*n*, %)	7 (13.5)	21 (3.1)	14.194	0.002*
AD, yes (*n*, %)	7 (13.5)	16 (2.3)	19.667	0.001*
Eye disease yes (*n*, %)	7 (13.5)	40 (5.9)	4.652	0.041*
COPD, yes (*n*, %)	8 (15.4)	10 (1.5)	39.127	<0.001*
CKD, yes (*n*, %)	7 (13.5)	13 (1.9)	24.34	<0.001*
Chronic liver disease, yes (*n*, %)	1 (1.9)	7 (1.0)	0.36	0.548

*p < 0.05.

**Table 2:** Baseline characteristics of patients in the training set and validation set.

Variable	Training set (*n* = 513)	Validation set (*n* = 221)	Statistical value	*p* value
Contralateral hip fracture (*n*, %)	31 (6)	21 (9.5)	2.808	0.094
Gender (*n*, %)			2.774	0.096
Male	139 (27.1)	174 (78.7)		
Female	374 (72.9)	47 (21.3)		
Age (years)	79 (70, 84)	79 (73, 84)	−1.689	0.091
Hospitalization time (d)	9 (7, 13)	9 (7, 13)	−0.418	0.676
Place of residence (*n*, %)			0.006	0.939
City	389 (75.8)	167 (75.6)		
Rural	124 (24.2)	554 (24.4)		
ASA class (*n*, %)			0.792	0.851
1	5 (0.5)	1 (0.5)		
2	185 (36.1)	84 (38)		
3	292 (56.9)	124 (56.1)		
4	31 (6)	12 (5.4)		
Height (m)	1.58 (1.55, 1.65)	1.57 (1.55, 1.62)	−1.202	0.23
Weight (kg)	60 (52, 68)	60 (42.5, 68)	−0.947	0.344
BMI (kg/m^2^)	23.4 (20.8, 26.6)	23.7 (21.5, 26.7)	−1.465	0.143
Fracture type (*n*, %)			0.905	0.372
Intertrochanteric fracture	215 (41.9)	101 (45.7)		
Femoral neck fracture	298 (58.1)	120 (54.3)		
Surgical approach (*n*, %)			3.063	0.382
Closed reduction internal fixation	212 (41.3)	101 (45.7)		
Hemiarthroplasty	125 (24.4)	58 (26.2)		
Total hip arthroplasty	124 (24.2)	46 (20.8)		
Open reduction internal fixation	52 (10.1)	16 (7.2)		
WBC (10^9^/L)	8.08 (6.46, 9.65)	8.23 (6.43, 10.00)	−0.727	0.468
Hb (g/L)	121 (107, 132)	117.33 ± 19.72	−1.145	0.252
PLT (10^9^/L)	186 (153, 227)	186 (155.5, 225.5)	−0.001	0.999
NEU (10^9^/L)	6.03 (4.66, 7.57)	6.15 (4.55, 7.61)	−0.24	0.811
LYM (10^9^/L)	1.19 (0.90, 1.55)	1.23 (0.97, 1.60)	−1.674	0.094
NLR	5.03 (3.42, 7.28)	4.76 (3.31, 6.83)	−1.056	0.291
PLR	159.34 (120.04, 216.6)	147.93 (109.47, 198.61)	−1.988	0.047*
SII	939.5 (595.31, 1476.45)	873.43 (606.09, 1339.62)	−0.88	0.379
Calcium (mmol/L)	2.2 (2.13, 2.27)	2.19 (2.12, 2.27)	−1.383	0.167
Phosphorus (mmol/L)	1.11 (1.00, 1.25)	1.11 (1.00, 1.26)	−0.184	0.854
Calcium-phosphorus product	30.59 ± 5.61	30.01 (26.79, 34.60)	−0.211	0.833
Potassium (mmol/L)	3.8 (3.52, 4.10)	3.83 (3.56, 4.10)	−0.653	0.514
Sodium (mmol/L)	139 (136, 141)	139 (136,141)	−0.294	0.769
FBG (mmol/L)	6.53 (5.75, 7.74)	6.41 (5.53, 7.74)	−1.078	0.281
ALB (g/L)	37.3 (34.4, 39.8)	37.1 (34.05, 39.8)	−0.775	0.439
ALP (U/L)	78 (66, 98)	76 (65.95, 93)	−1.106	0.269
Cr (μmol/L)	60 (51, 75)	60 (50, 77)	−0.475	0.635
BUN (mmol/L)	6.2 (4.87, 7.85)	6.18 (4.81, 8.23)	−0.081	0.935
Hypertension, yes (*n*, %)	241 (47)	106 (48)	0.06	0.81
Heart disease, yes (*n*, %)	109 (21.2)	50 (22.6)	0.173	0.697
Diabetes, yes (*n*, %)	120 (23.4)	54 (24.4)	0.093	0.777
Neurovascular disease, yes (*n*, %)	93 (18.1)	39 (17.6)	0.024	0.917
PD, yes (*n*, %)	21 (4.1)	7 (3.2)	0.361	0.676
AD, yes (*n*, %)	20 (3.9)	3 (1.4)	3.286	0.103
Eye disease, yes (*n*, %)	35 (6.8)	12 (5.4)	0.5	0.517
COPD, yes (*n*, %)	13 (2.5)	5 (2.3)	0.048	0.827
CKD, yes (*n*, %)	11 (2.1)	9 (4.1)	2.166	0.214
Chronic liver disease, yes (*n*, %)	6 (1.2)	2 (0.9)	0.1	0.751

*p < 0.05.

### 3.2 Screening for predictive factors

**Figure 1:**
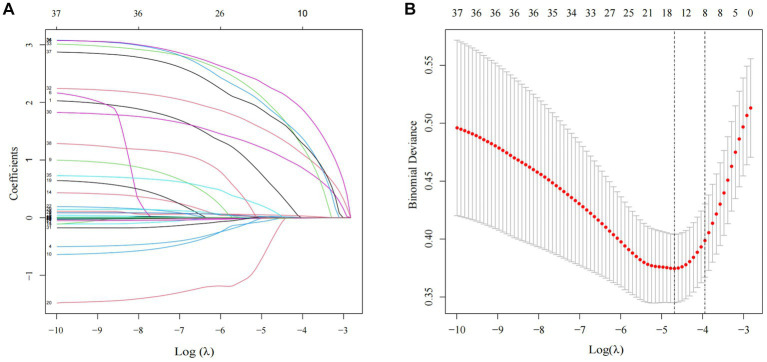
Clinical characteristics of LASSO regression analysis. **(A)** Predictor coefficient graphic. Each colored solid line in the figure represents a variable with decreasing variable coefficients as Log (λ) increases and part of the variable coefficients become zero. **(B)** Lasso regression model cross-validation graph. The dotted vertical line on the left and right of the graph represents the value of log (λ. min) and (λ.1se).

The Lasso regression analysis revealed that when lambda. Min (0.009215216) was selected as the best lambda value, and eight variables with nonzero coefficients were screened out to be the critical variables associated with the subsequent contralateral hip fracture, including age, Hb, heart disease, neurovascular disease, PD, AD, COPD, and CKD, as shown in Figure 1. Subsequently, the optimal model variables were further screened using logistic regression analysis, which demonstrated that age (OR, 1.09, 95% CI, 1.032–1.156, *p* = 0.003), Hb (OR, 0.981, 95% CI, 0.963–0.999, *p* = 0.049), heart disease (OR, 4.462, 95% CI, 2.211–9.174, *p* < 0.001), neurovascular disease (OR, 6.049, 95% CI, 2.947–12.625, *p* < 0.001), PD (OR, 11.618, 95% CI, 3.583–36.299, *p* < 0.001), AD (OR, 12.14, 95% CI, 3.545–39.225, *p* < 0.001), COPD (OR, 18.591, 95% CI, 5.492–63.181, *p* < 0.001), CKD (OR, 8.29, 95% CI, 2.339–28.117, *p* = 0.001) were statistically significant independent risk factors. The details can be seen in Table 3.

**Table 3:** Univariate and multivariate logistic regression analyses of variables relating to subsequent contralateral hip fracture.

Variables	Univariate-analysis		Multivariate-analysis	
	OR (95% CI)	*p* value	OR (95% CI)	*p* value
Age	1.091 (1.048–1.135)	<0.001	1.09 (1.032–1.156)	0.003
Hb	0.972 (0.959–0.985)	<0.001	0.981 (0.963–0.999)	0.049
Heart disease	4.907 (2.754–8.743)	<0.001	4.462 (2.211–9.174)	<0.001
Neurovascular disease	5.434 (3.037–9.722)	<0.001	6.049 (2.947–12.625)	<0.001
PD	4.896 (1.977–12.129)	0.001	11.618 (3.583–36.299)	<0.001
AD	6.475 (2.534–16.544)	<0.001	12.14 (3.545–39.225)	<0.001
COPD	12.218 (4.593–32.504)	<0.001	18.591 (5.492–63.181)	<0.001
CKD	8.005 (3.043–21.057)	<0.001	8.29 (2.339–28.117)	0.001

### 3.3 Nomogram construction and evaluation

**Figure 2:**
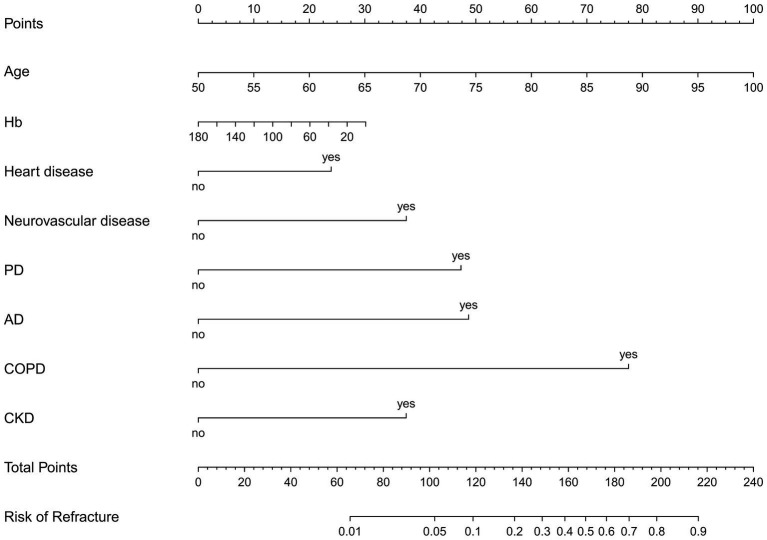
The nomogram predicts the risk of s subsequent contralateral hip fracture in elderly patients within 2 years after hip fracture surgery, based on age, Hb, heart disease, neurovascular disease, PD, AD, COPD, and CKD. Hb, hemoglobin; PD, Parkinson’s disease; AD, Alzheimer’s disease; COPD, chronic obstructive pulmonary disease; CKD, chronic kidney disease.

**Figure 3:**
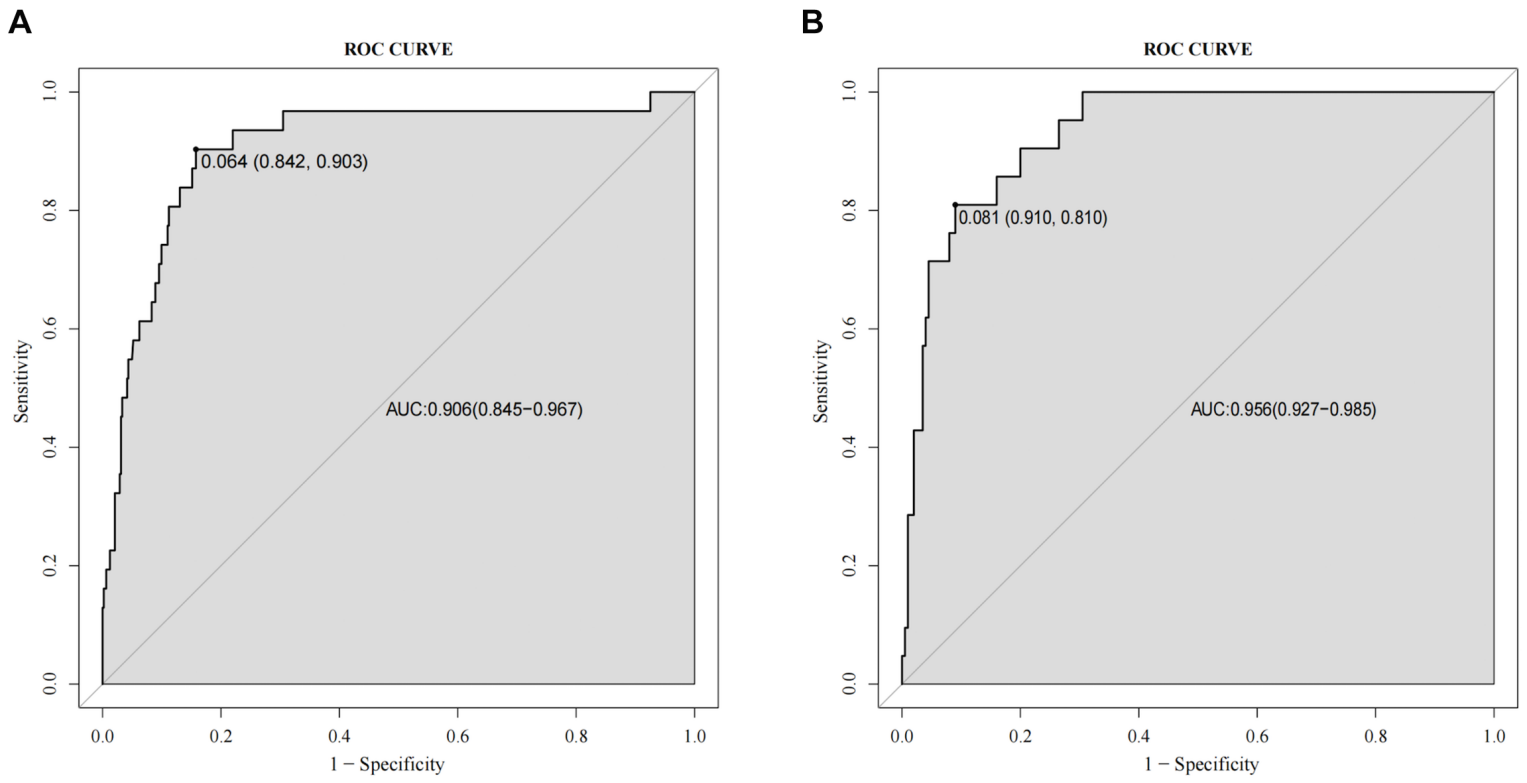
Comparison of the receiver operating characteristic curve (ROC) and the area under the curve (AUC) of the nomogram in the training set **(A)** and the validation set **(B)**.

**Figure 4:**
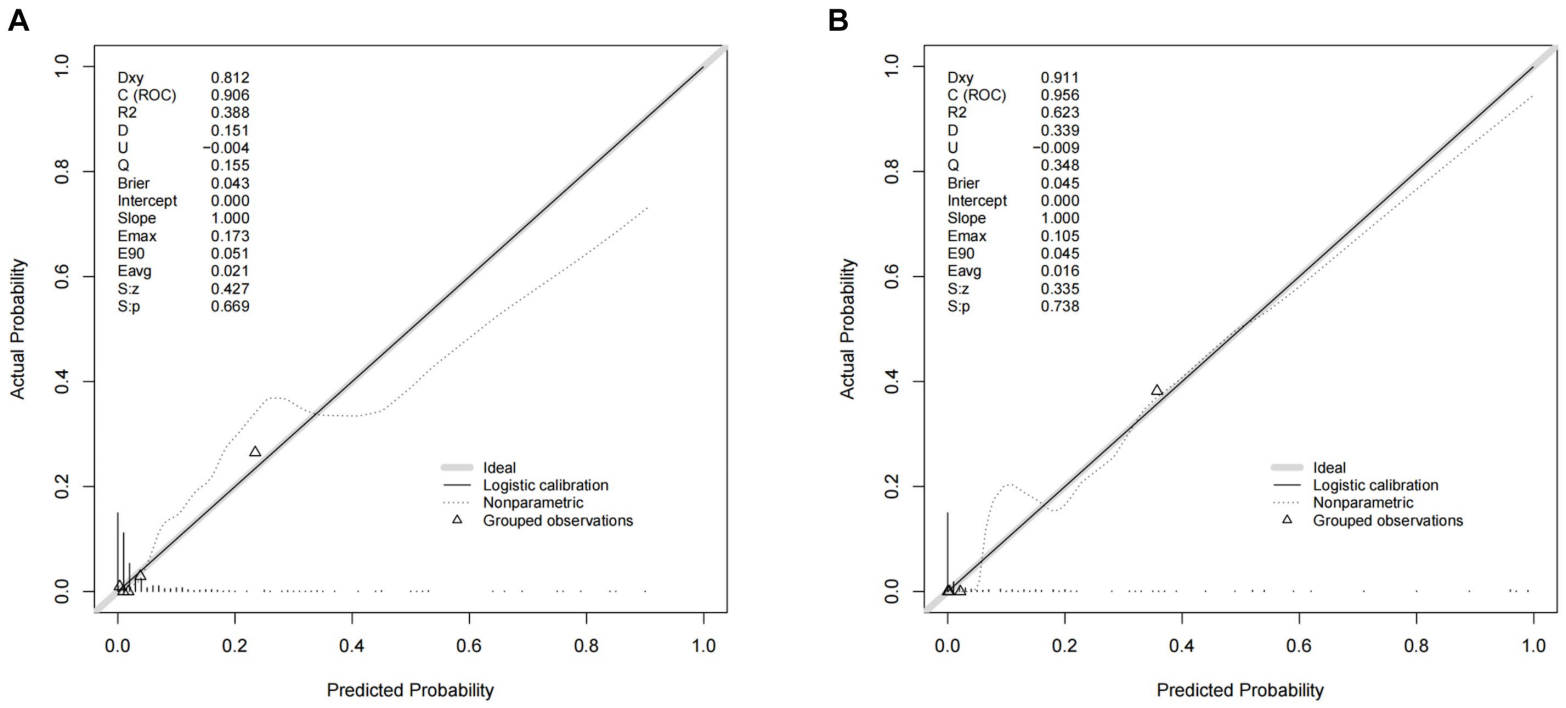
Comparison of the calibration curves of the nomogram in the training set **(A)** and the validation set **(B)**.

**Figure 5:**
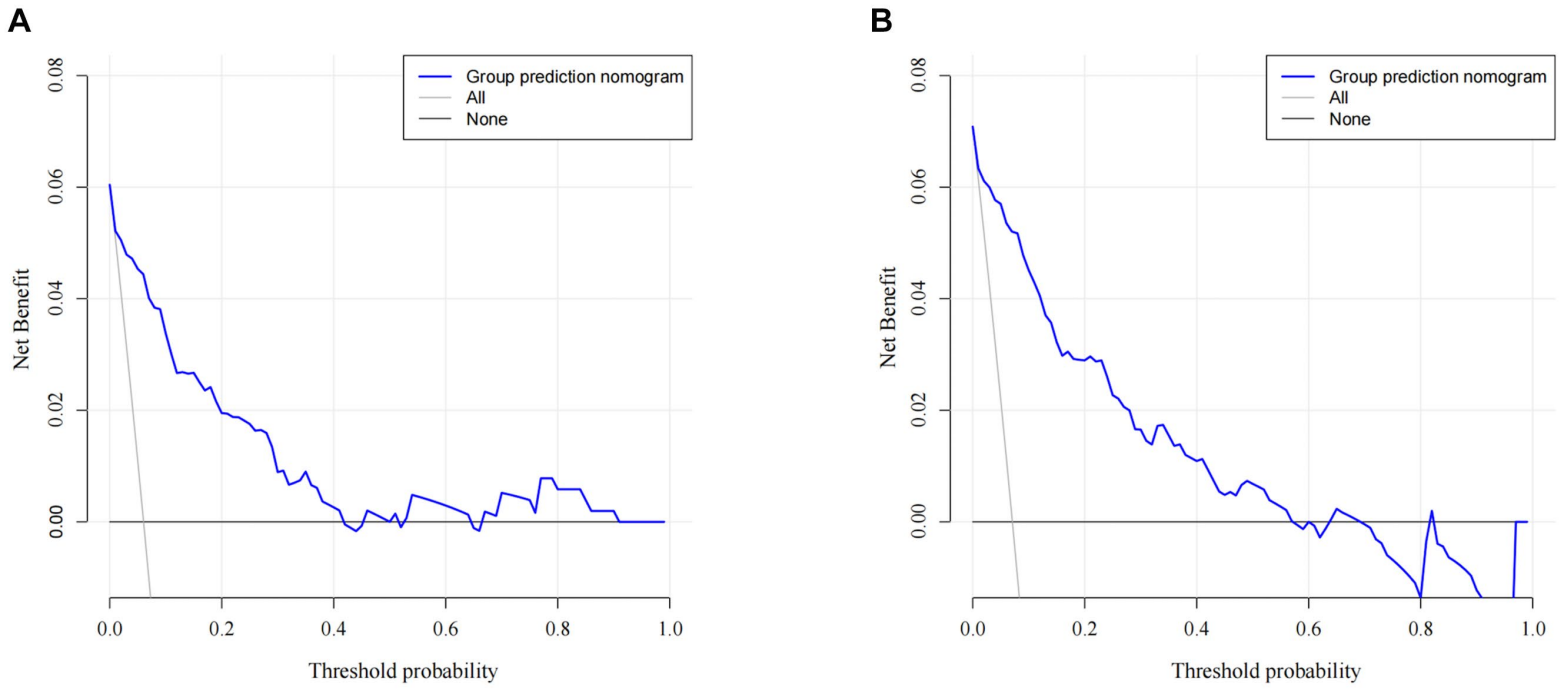
Comparison of the decision curve analyses (DCA) of the nomogram in the training set **(A)** and the validation set **(B)**.

A nomogram of the training set was constructed based on the eight optimal predictors screened by Lasso regression combined with logistic regression (Figure 2). The scores corresponding to the different categories of independent risk factors for each patient were calculated by summing the patient’s total score values, and the corresponding total score scales represented the probability of the patients suffering subsequent contralateral hip fractures. The nomogram model could be applied for incidence calculation. For assessing the nomogram’s discriminatory ability, we plotted the ROC curve for the training and validation sets and calculated the AUC. The model’s cut-off value was identified: 0.064 in the training set (specificity 0.842 and sensitivity 0.903); 0.081 in the validation set (specificity 0.91 and sensitivity 0.81). The AUC was 0.906 (95% CI, 0.845–0.967) and 0.956 (95% CI, 0.927–0.985) in the training and validation sets, separately (Figure 3), indicating that the prediction model has a great discriminatory performance. The Hosmer-Lemeshow test demonstrated no significant difference in the training and validation sets (*p* = 0.149 and 0.972, respectively). This demonstrated that the predictions of the model are similar to actual subsequent contralateral hip fracture incidence, illustrating the robust calibration ability of the nomogram (Figure 4). According to DCA in the training set, the nomogram brought positive net benefit with a threshold probability of 1–75%. The validation set showed a higher net benefit with a threshold probability of 1%–56% (Figure 5). The results demonstrated excellent clinical efficacy of the model and promising agreement consistency between the training and validation sets.

## 4 Discussion

This study for the first time established a predictive model for subsequent contralateral hip fractures after initial surgery. In the previous studies, the majority of risk factors were initially screened out by univariate analysis, followed by identifying independent risk factors by multivariate logistic regression analysis. Considering the interactions among these independent risk factors, biases such as multicollinearity and model overfitting were inevitable. Our study utilized the LASSO regression analysis with cross-validation to screen out the potentially associated risk factors, reducing the influence of multiple covariances on the ultimate prediction model and enhancing the model fitting performance. In our study, we found that age, Hb, heart disease, neurovascular disease, PD, AD, COPD, and CKD acted as independent risk factors. Consistently, a previous high-quality prospective cohort study has also provided related evidence (21).

As has been evidenced, old age can independently affect subsequent contralateral hip fractures (12, 14, 22). Our study shows that the odds ratio increases by 1.09 for each additional year after 60 years old. Analyzing the underlying reasons, generally elderly patients may be more likely to suffer from severe osteoporosis and various medical comorbidities. A prospective study revealed that patients with anemia have an enhanced risk of hip fracture (23). As has been demonstrated by Sim et al., preoperative anemia (Hb < 10.0 g / dL) is related to worse physical function and life quality postoperatively, which increased the risk of falls (24). Anaemia was commonly considered to be associated with osteoporosis and sarcopenia in the elderly (25), whereas muscle mass and function loss enhance fragility fracture risk through increasing fall risk (26).

In our study, heart disease can also independently affect contralateral hip refracture, which was in agreement with previous findings (27, 28). A large analysis conducted among 113,600 individuals revealed that atrial fibrillation history enhances subsequent hip fracture risk (29). In a previous systematic review, the odds ratio for heart failure is 1.3 (95% CI, 1.00–1.78) for contralateral hip refracture in elderly patients (30). However, we referred to coronary heart disease, arrhythmias, heart failure, and myocardial infarction collectively as heart disease in our study, which may lead to selection bias. COPD was associated with osteoporosis and hip fractures (12, 31). Analyzing the reasons, patients with COPD received long-term glucocorticoid therapy, which contributed to osteoporosis, and COPD patients had lower physical activity and were consequently deficient in muscle strength and endurance, predisposing to fragility fractures. Patients with glomerular filtration rates (GFR) of 30 to 44 and 15 to 29 mL/min / 1.73m^2^ have a higher risk of hip fracture than those with GFR > 60 mL/min/1.73m^2.^ Consistent results were also evidenced in our study (32). Vitamin D metabolism is abnormal in CKD patients, as manifested by decreased calcidiol levels due to short daylight exposure and low vitamin D intake, and reduced calcitriol synthesis due to decreased renal function, increasing osteoporosis risk in CKD patients (33).

It has been revealed that the association between neurovascular disease can affect subsequent hip fractures and may be related to increased fall risk due to compromised balance and abnormal proprioception, which was similar to our findings (12, 34, 35). Most hip fractures occur following a fall and that impaired balance and postural instability in patients with PD contribute to the increased risk of falls (36). Nam JS et al. revealed that the risk of hip fracture was about approximately 2-fold higher in people with PD than in the control group, which bolstered our research results (37). AD was a degenerative change in the central nervous system, characterized by progressive loss of memory and cognition. The majority of Alzheimer’s patients had balance impairments and gait deficits, increasing fall risk (38). In addition, some studies demonstrated an association between cognitive decline and hip fracture risk (39).

Unfortunately, there remained a lack of tools to assess the risk of contralateral hip refracture after surgery. The Fracture Risk Assessment Tool (FRAX), a predicting tool based on individual patient models, integrates clinical risk factors and bone mineral density (BMD) at the femoral neck (40). However, the FRAX was primarily designed for predicting an initial fracture risk and the application of its capability in predicting subsequent contralateral hip fractures remains to be further validated (41, 42). QFractureScores was developed to assess an individual’s risk of an osteoporotic fracture within 10 years, based on a prospective cohort study in England, with 25 components including comorbidities (21). However, QFractureScores was also designed for initial fracture assessment and contained excessive variables that might not facilitate clinical application (43).

We first developed a nomogram prediction model for subsequent contralateral fractures in elderly patients within 2 years after hip surgery that included demographic variables, clinical characteristics, and laboratory examination, ultimately identifying the eight optimal predictors. Our nomogram prediction model exhibited good discriminability. Additionally, the nomogram was well-calibrated both in the training and validation sets, indicating good consistency between the actual subsequent contralateral hip fracture incidence and the predicted probability. The DCA confirmed the nomogram model’s excellent clinical utility.

However, there are some limitations. Firstly, all the analyzed data in this retrospective study were limited by the medical records, which can cause methodological bias. Secondly, this study was single-centered without external validation. All patients were included from the same senior trauma center, which meant more severe comorbidities and advanced disease than in other minor medical institutions. Third, the patient size included in our study was relatively small, thus, a large-sample and multi-center clinical study to optimize the clinical prediction model of subsequent contralateral hip fractures after hip fracture surgery will be necessary.

## 5 Conclusion

Taken together, we developed and validated a nomogram prediction model incorporating age, Hb, heart disease, neurovascular disease, PD, AD, COPD, and CKD to predict the occurrence of subsequent contralateral hip fracture within 2 years after operation in the elderly. We expect that this straightforward and visual predictive model will provide clinicians with precise references for appropriate perioperative management and rehabilitation education following initial hip surgery for their patients.

## Data availability statement

The raw data supporting the conclusions of this article will be made available by the authors, without undue reservation.

## Ethics statement

The studies involving humans were approved by the Ethics Committee of the First Affiliated Hospital of Dalian Medical University. The studies were conducted in accordance with the local legislation and institutional requirements. The participants provided their written informed consent to participate in this study. Written informed consent was obtained from the individual(s) for the publication of any potentially identifiable images or data included in this article.

## Author contributions

JL: Data curation, Formal analysis, Investigation, Methodology, Software, Writing – original draft, Writing – review & editing. JZ: Writing – original draft, Writing – review & editing. ZL: Formal analysis, Supervision, Writing – review & editing. XT: Investigation, Supervision, Writing – review & editing.
